# Supplementary material for: Phylogenetic and functional distinctiveness explain alien plant population responses to competition
Source: Proc Biol Sci. 2020 Jul 1;287(1930):20201070. doi: 10.1098/rspb.2020.1070 (PMC7423470; doi:10.1098/rspb.2020.1070)
Supplement: Appendices 1–3 [file rspb20201070supp1.pdf]

SUPPLEMENTARY MATERIAL

Appendix 1: Supplementary tables and data

Table S1.1: Effect sizes, biomass removed, site-level species richness, and % cover of each the 14 focal species

Focal species	*Effect size of competitor removal*	*Standardized biomass removed*	*Site-level species richness*	*% Cover of focal species*
Ailanthus altissima	0.429	0.016	62	9.26
Alliaria petiolata	1.39	29.011	35	2.8
Carduus nutans	1.30	85.509	55	17.7
Draba verna	0.254	36.923	26	11.07
Euonymus alatus	0.00593	0.019	50	14.13
Kummerowia striata	3.00	2644.356	13	13.3
Lepidium campestre	1.16	39.477	32	0.6
Lespedeza cuneata	0.386	0.104	27	65.17
Ligustrum obtusifolium	0.0263	0.009	20	44.6
Lonicera maackii	-0.0309	0.013	64	24.91
Perilla frutescens	-0.22	12.827	30	9.8
Potentilla recta	0.3	272.133	28	6.58
Thlaspi perfoliatum	-0.0971	51.128	30	24.45
Verbascum thapsus	1.155	50.296	27	18.1

Appendix 2: Competitor removal biomass methods and testing for phylogenetic biases

We conducted statistical tests to examine whether plants with low or no responses to competitor removal were actually dominant competitors, rather than this resulting from some artefact of our methods or site selection. The first test investigated whether the site's species richness had a significant impact on the effect size of competition. One might expect that focal species with sites that have fewer species might have less response to competition simply because there are fewer competitors. However, this is not the case (Figure S2.1). Furthermore, the standardized values of competitor removal biomass declined with increasing species richness (Figure S2.2), suggesting that dominance of the focal species caused many co-occurring species to become rare. Indeed, sites with a higher abundance of focal species were not necessarily less species rich (Figure S2.3), but there was a negative, though insignificant relationship between non-focal species abundance and focal species abundance (Figure S2.4). This supports the conclusion that dominant focal species caused others to become more rare, rather than sampling bias that caused us to select sites without competitors.

Our next tests for biases focus on the way the biomass covariate was included in our models. In the body of the paper, the biomass covariate is calculated as average biomass per plot per gram of focal species biomass. This accounts for both the differing size of the plots and for the fact that our focal species differed in size. 50g of biomass is unlikely to have the same competitive effect on *Ligustrum* obtusifolium, a small tree, as it does on *Kummerowia striata*, a small herb. First, we fit models that do not account for competitive asymmetry between any focal species and their resident communities.Prior theoretical and empirical work has shown that asymmetric competition is prevalent across many types of ecosystems and can affect results if not properly considered (Weiner et al 2001, Pretzsch & Biber 2010). To be safe, we tested another ways of modeling the demographic results. We used residuals from a regression of the effect size of competition on the size standardized competitor biomass covariate as the response variable for the regression bootstrapping described in the main text. We found similar patterns as reported in the manuscript text (significant, negative relationships between phylogenetic distinctiveness and invasiveness at the local scale, non-significant relationships at regional scale, Figure S2.5). We then explored the effects of modeling our results without accounting for the biomass removed from our plots.

$$\frac{\frac{Biomass\ of\ competitors\ (g)}{Plot\ Area\ (m^2)}}{Biomass\ of\ focal\ species\ (g)} \quad (1)$$

Kummerowia striata appears to drive a substantial portion of the negative relationship between distinctiveness and invasiveness, so we re-ran all analyses omitting it to see if the effects were still present (Figures S2.6).

Finally, we tested whether our results were robust to choice of phylogeny by re-running all core analyses presented in the main text with two other phylogenies

obtained from Smith & Brown (2018). Our results also do not change qualitatively when using these larger trees (Figures 2.7-10).

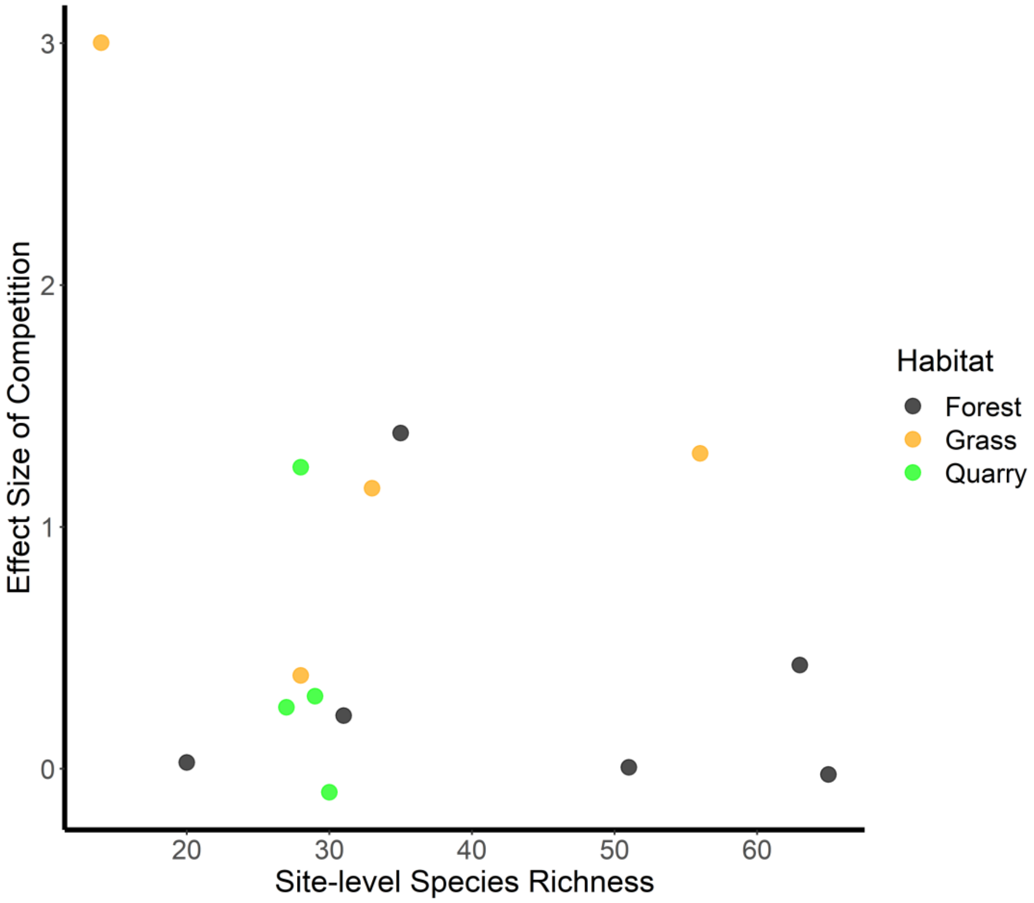

Figure S2.1: Site-level species richness does not predict response to competition (linear model, $F = 1.38$, $R^2_{adj} = 0.028$, $p = 0.2628$).

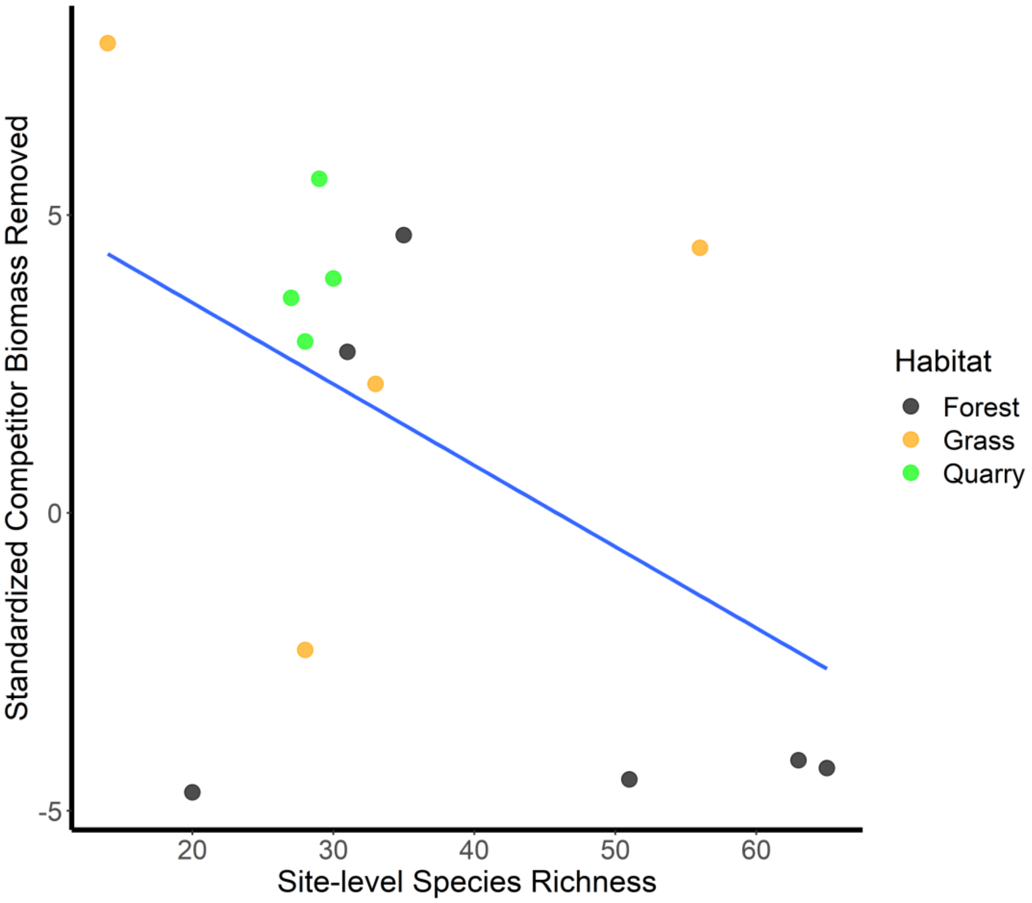

Figure S2.2: Standardized competitor removal biomass declines with increasing species richness (linear model, $F = 3.987, R^2_{adj} = 0.1868, p = 0.069$).

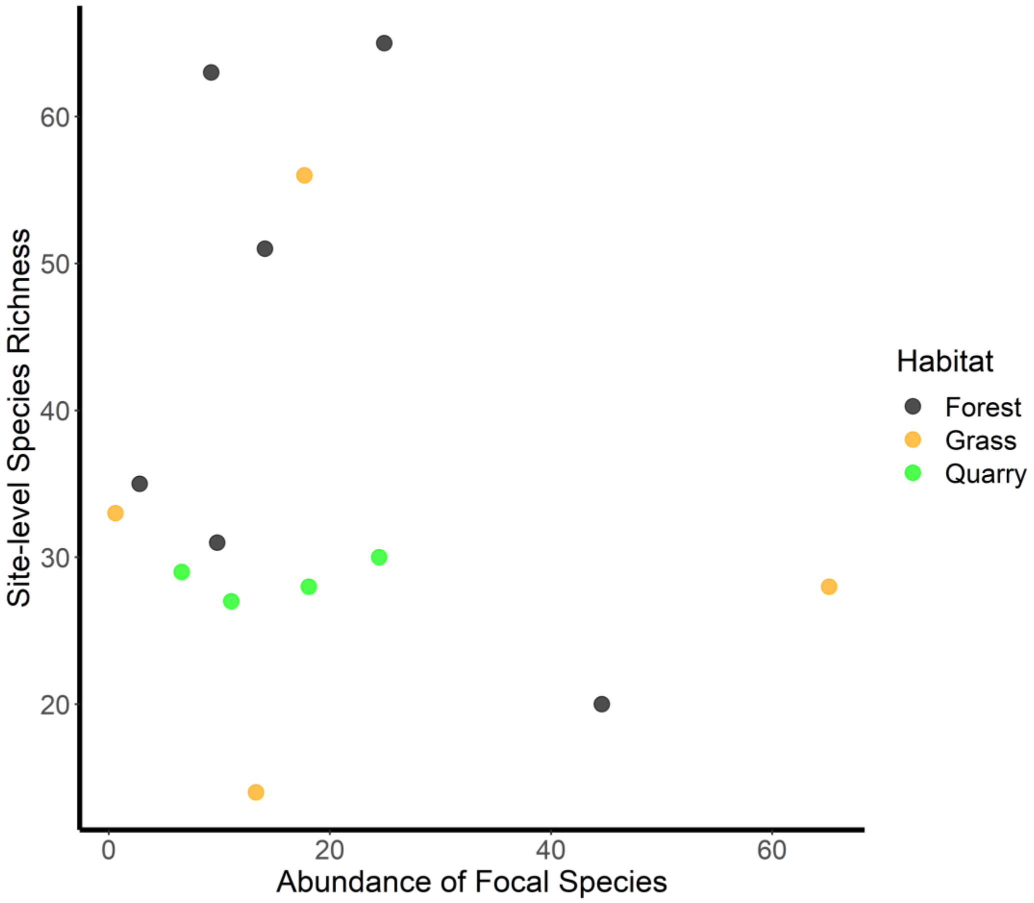

Figure S2.3: Abundance of focal species did not drive species richness at our sites (linear model, $F = 0.340$, $R^2_{adj} = -0.053$, $p = 0.5704$).

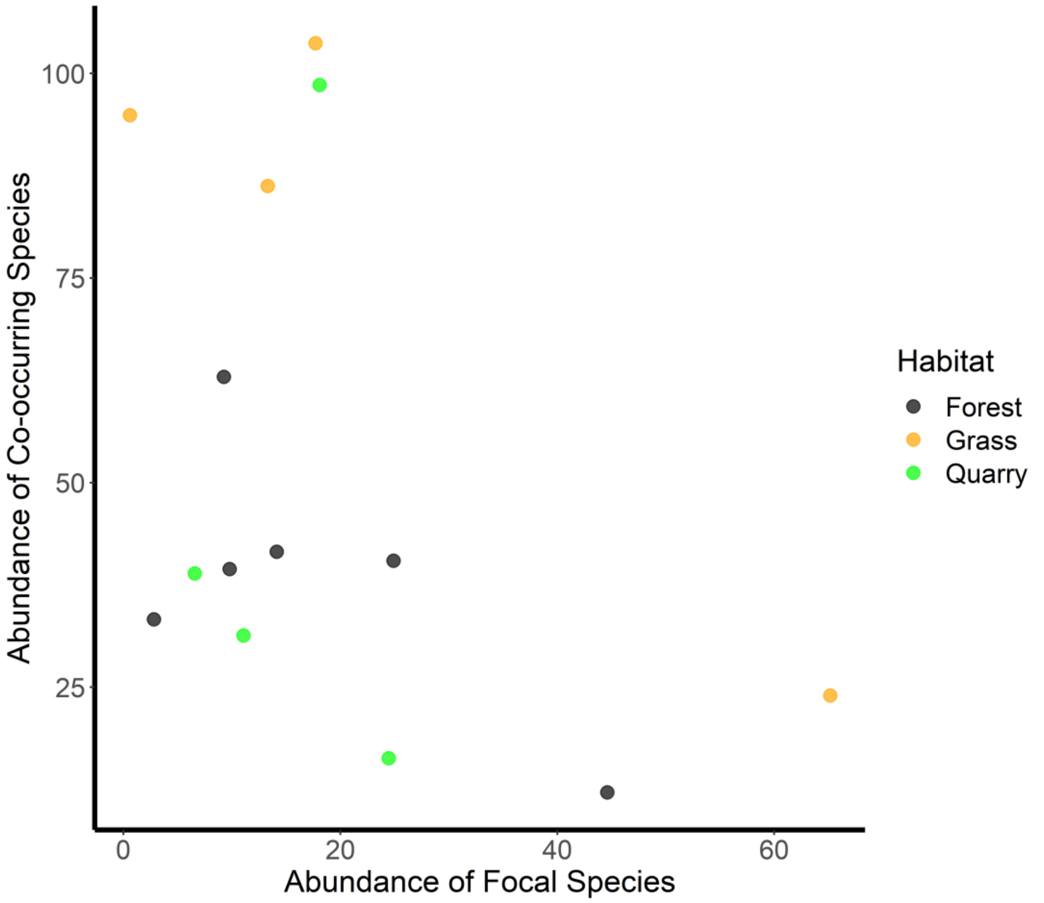

Figure S2.4: Non-focal species abundance is seemingly unrelated to increasing focal species abundance (linear model: $F = 2.552$, $R^2_{adj} = 0.107$, $p = 0.1361$).

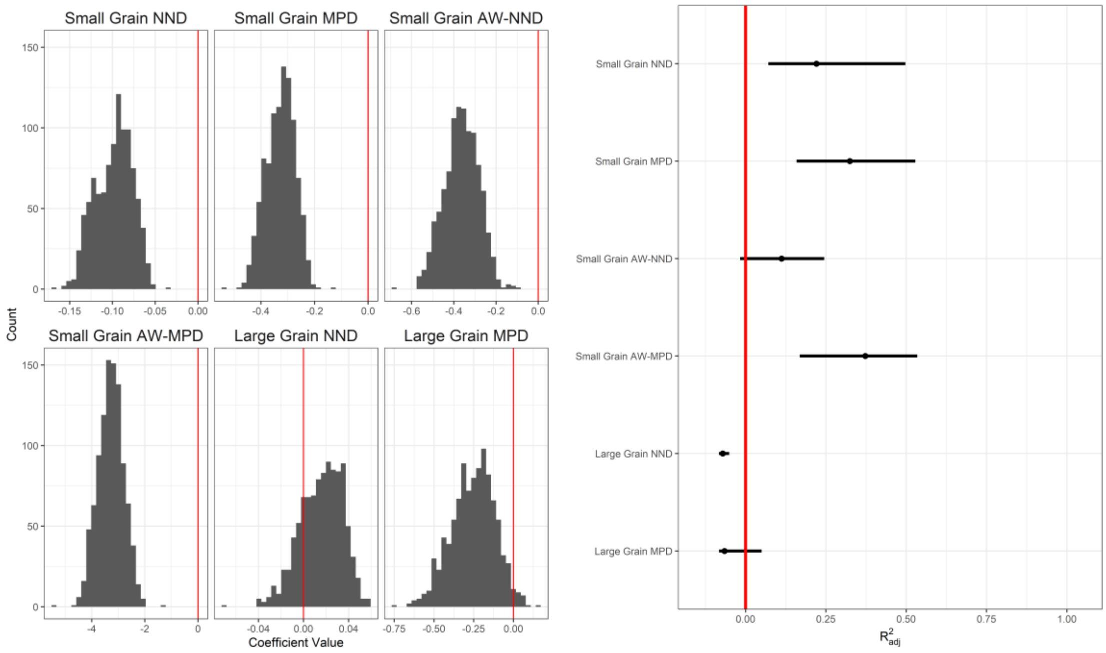

Figure S2.5: The residuals of a regression of the observed effect size of competition on competitor biomass were also used as response variables in regressions with phylogenetic distinctiveness. The qualitative results do not change.

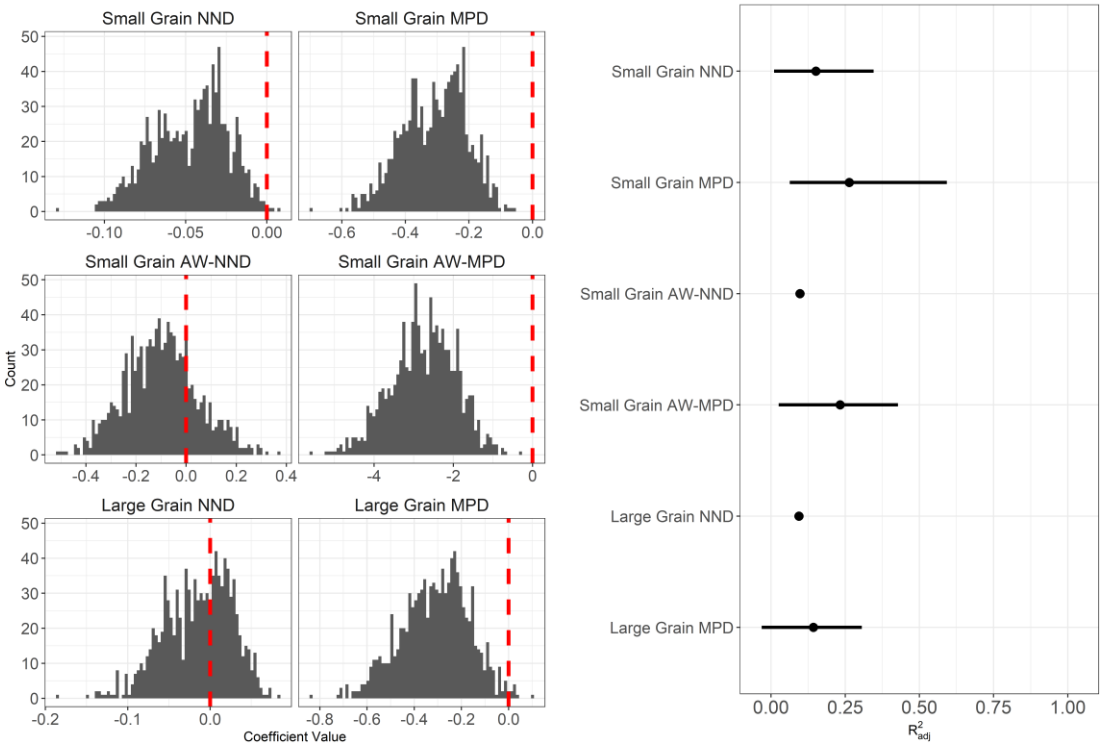

Figure S2.6: Regression without *Kummerowia striata*, the focal sprecies that was least novel and most affected by the competitor removal treatment, result in similar relationships as those with competitor removal biomass for MPD, but not NND.

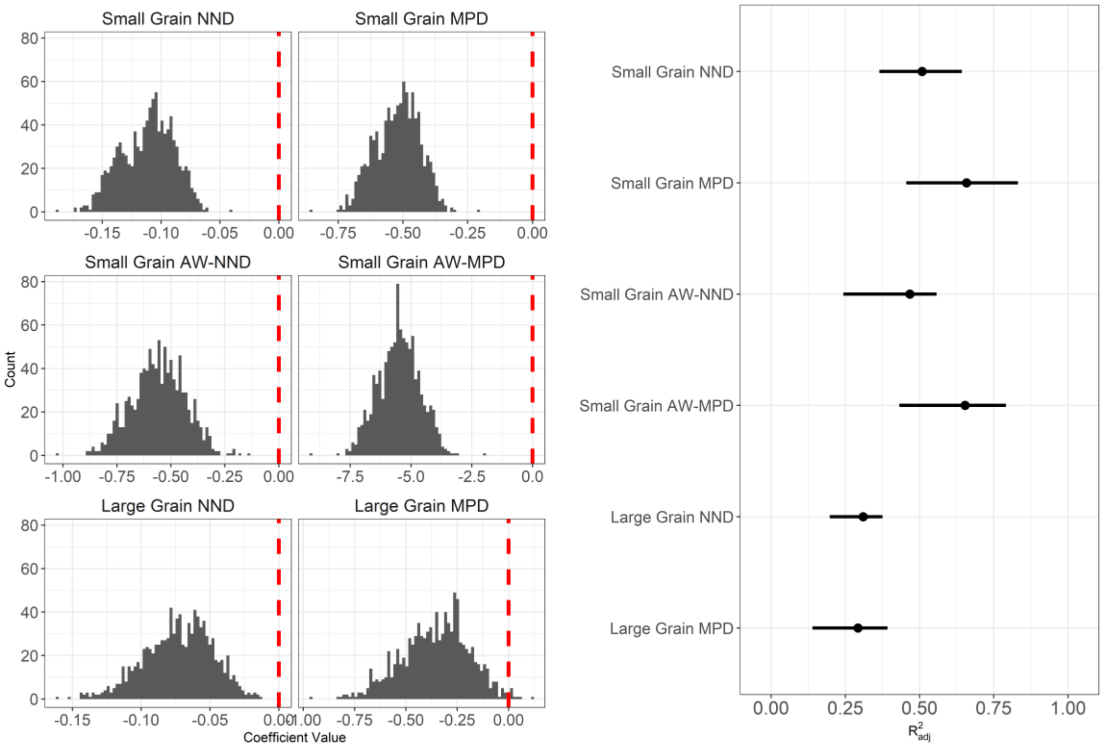

Figure S2.7: The results using the GenBank-only phylogeny from Smith & Brown (2018) did not alter the direction or explanatory power of the phylogeny-only models.

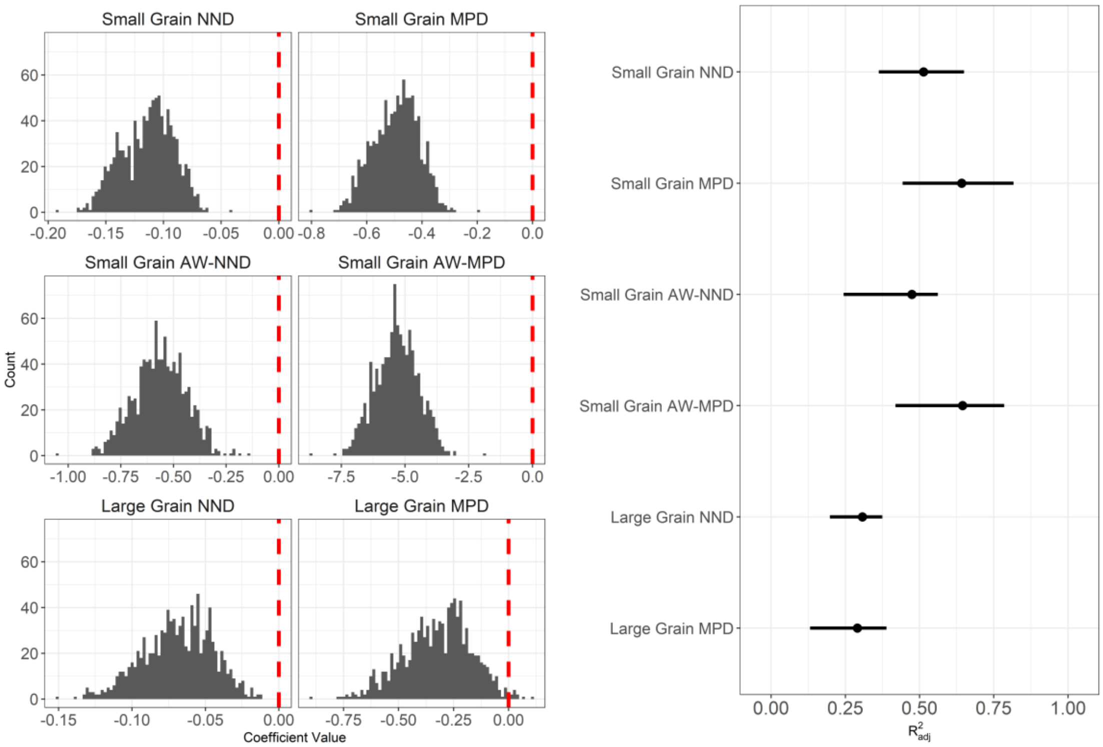

Figure S2.8: The results using the combined Genbank – Open Tree of Life phylogeny from Smith & Brown (2018) did not alter the direction or explanatory power of the phylogeny-only models.

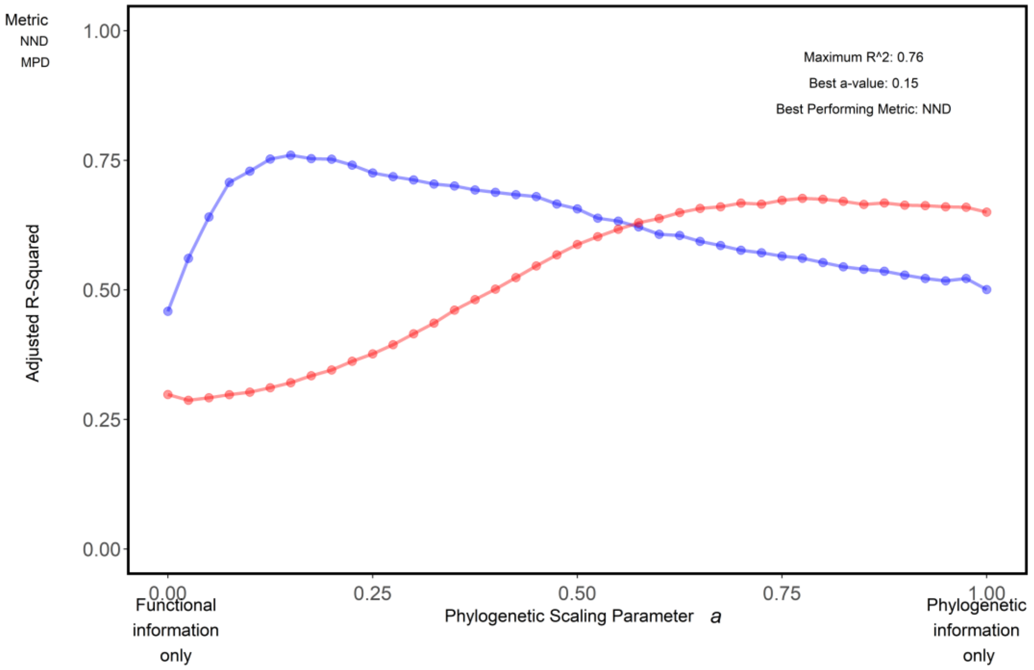

Figure S2.9: The shape of the response curves to *a* is qualitatively similar for the GenBank only tree and the Zanne et al. tree.

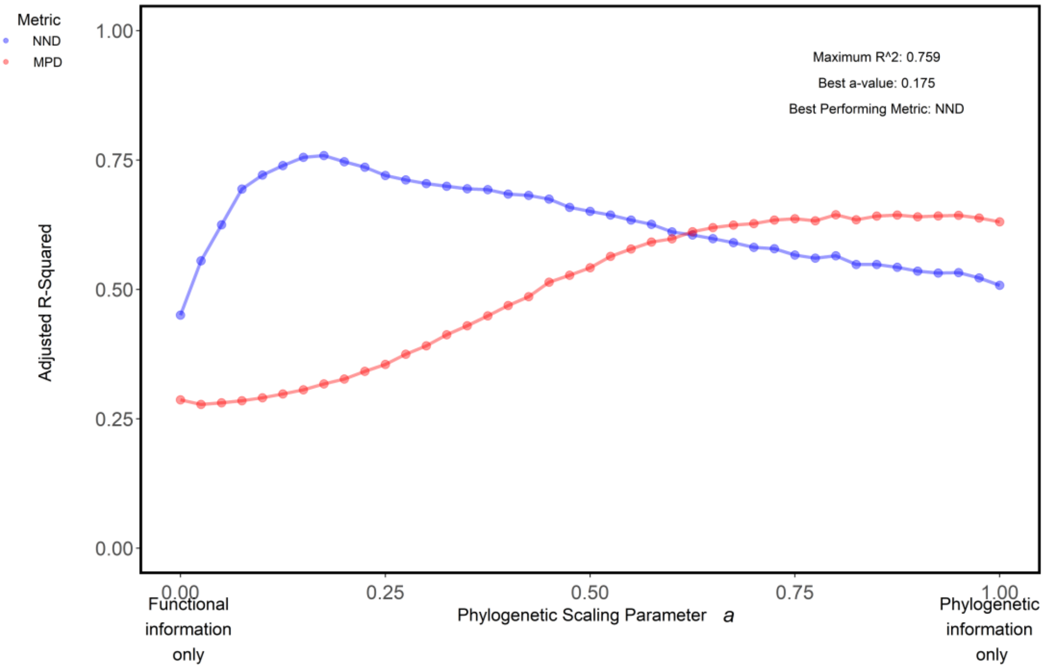

Figure S2.10: The shape of the response curves to *a* is very similar for the combined Genbank – Open Tree of Life tree.

Appendix 3: Definitions, extended methods and results for functional traits

We collected functional trait information for 119 plant species at Tyson Research Center and Shaw Nature Reserve between spring of 2013 and fall of 2015. However, three species were not included in our analysis for the following reasons: *Euphorbia maculate* and *Stenaria nigricans* had leaves that were too small to measure SLA and toughness. Thus, comparisons to other species using only continuous traits in our functional models and functional-phylogenetic models were not meaningful. However, we have included all of the above mentioned data in the published data set should any other researchers find this information informative in their own analyses.

Chlorophyll content (correlated with leaf toughness, $r = 0.70$) and wet/dry ratio (correlated with SLA, $r = 0.57$) were excluded from all analyses after running pairwise Pearson correlation tests to avoid including redundant information.

Growth form (2 tiered): Level 1: Woody/Herbaceous, Level 2: Vine, stemmed herb, see Perez-Harguindeguy 2013 for details of levels.

Dispersal syndrome consisted of the following levels: unassisted, wind, exozoochory, endozoochory, ballistic dispersal, animal hoarding, myrmecochory, and water. Each species was assigned at least one of these categories and potentially up to three if sources indicated they were all utilized by the species.

Clonality was defined as whether or not the species has vegetative reproductive organs above or below ground. If there were any reports of clonal reproduction, this was scored as 1. Otherwise, it was scored as 0.

Wood density information: For the 116 plant species in our reduced dataset, we included information on species mean values when possible (*N* = 13 species), and accepted information that represented the mean for genus when species level information was unavailable (*N* = 18 species). We omitted any data that represented an average value for taxonomic ranks higher than genus (*N* = 84 species). The library contained no information for one woody focal species (*L. maackii*), so we used information collected by other researchers at TRC (Spasojevic et al 2016).

Mean coverage of community level abundance per trait across all three habitat types is in Table S3.4, and the proportion of the community covered by our trait sampling by habitat type is shown in Figure S3.1. Proportion of coverage was estimated by computing the total community abundance, the total community abundance for which a given trait had a value, and then dividing second value by the first value.

Finally, we tested whether combinations of traits without any phylogenetic information can explain the effect size of competition. Figures S3.2-11 show results for combinations of traits with at least one significant relationship out of the 6 different metrics we computed. We found no combinations were as consistent or predictive as our integrated functional-phylogenetic metric, or phylogeny alone.

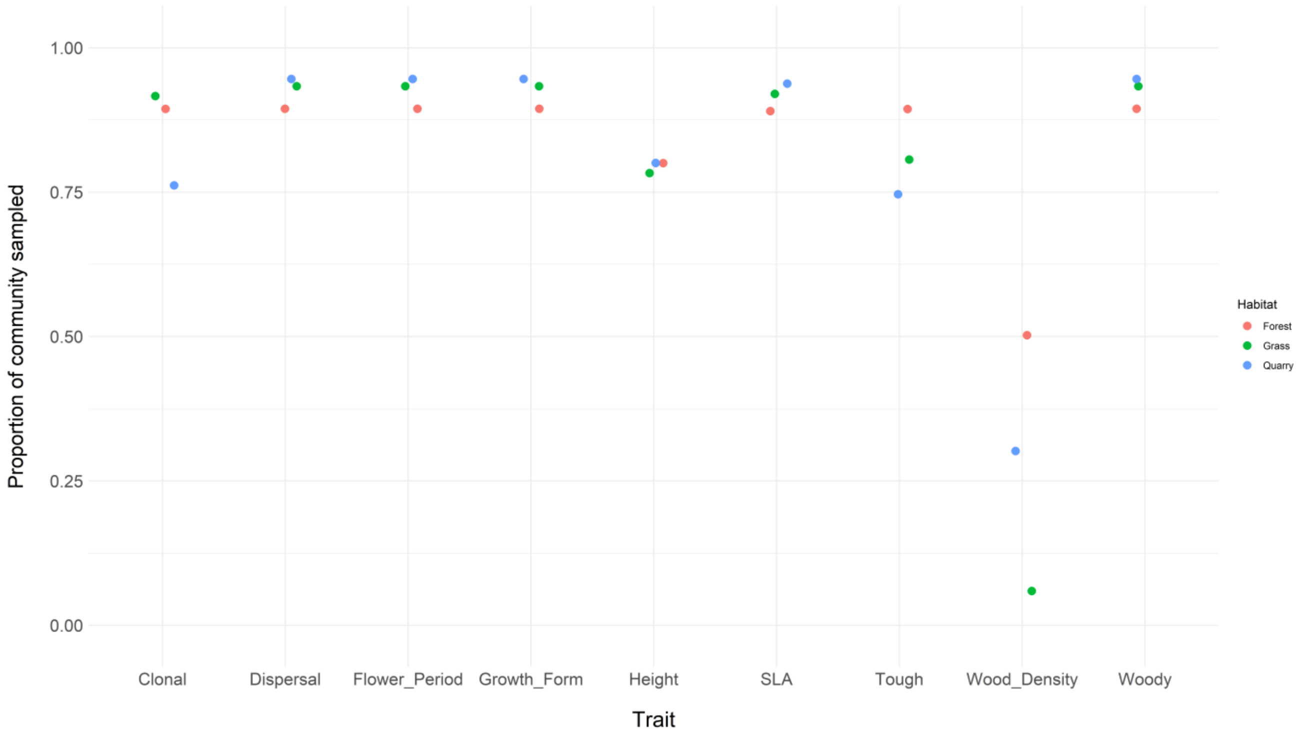

Figure S3.1: The proportion of each community that was sampled for each trait in our analysis. Blue dots indicate rocky outcropping/quarry habitat types, green dots indicate grassland habitat types, and red dots indicate forest habitat types. All wood density data comes from the BIOMASS R package (Rejou-Mechain et al. 2016).

Table S3.1: Results of tests for phylogenetic signal in our functional traits using the Zanne et al. 2013 tree. Significance levels for test statistics: *** $p \leq 0.001$, ** $p \leq 0.01$, * $p \leq 0.05$, + $p \leq 0.1$

	Data type	*Test*	*N*	*Value*	*p-value Brownian Motion*	*p-value random*
Continuous traits						
SLA	Continuous	Blomberg's K	110	0.1079	0.039*	
Height	Continuous	Blomberg's K	88	0.8109	0.002**	
Leaf toughness	Continuous	Blomberg's K	105	0.0416	0.328	
Wood density	Continuous	Blomberg's K	34	0.6014	0.007**	
Circular traits						
Flower Period	Circular	Mantel Test	116	0.0174	0.299	
Growth forms						
Stemmed Herb	Binary	Phylogenetic D	116	-0.1588	0.772	0***
Tree	Binary	Phylogenetic D	116	-0.0426	0.565	0***
Rosette	Binary	Phylogenetic D	116	-0.2345	0.789	0***
Vine	Binary	Phylogenetic D	116	0.6689	0.06+	0.117
SubShrub	Binary	Phylogenetic D	116	6.0974	0.162	0.817
Shrub	Binary	Phylogenetic D	116	0.2271	0.348	0.026*
Elongated Leafy Rhizomatous	Binary	Phylogenetic D	116	-0.0782	0.581	0***
Nitrogen Fixer	Binary	Phylogenetic D	116	-1.7008	1	0***
Dispersal mechanisms						
Subterranean	Binary	Phylogenetic D	116	-4.448	0.708	0.215
Unassisted	Binary	Phylogenetic D	116	0.7253	0***	0.025*
Wind	Binary	Phylogenetic D	116	0.483	0.01**	0***
ExoZoochory	Binary	Phylogenetic D	116	0.416	0.076+	0***
Ballistic	Binary	Phylogenetic D	116	0.3237	0.214	0.004**
EndoZoochory	Binary	Phylogenetic D	116	0.2743	0.119	0***
Hoarding	Binary	Phylogenetic D	116	-0.3601	0.672	0.005**
Myrmecochory	Binary	Phylogenetic D	116	1.6342	0.047*	0.826
Water	Binary	Phylogenetic D	116	0.9045	0.033*	0.397
All traits combined	Gower Distance Matrix	Mantel Test	116	0.2389	0.001***	

Table S3.2: Results of tests for phylogenetic signal in our functional traits using the combined OTL and Genbank tree. Significance levels for test statistics: *** $p \leq 0.001$, ** $p \leq 0.01$, * $p \leq 0.05$, + $p \leq 0.1$

	Data type	*Test*	*N*	*Value*	*p-value Brownian Motion*	*p-value random*
Continuous traits						
SLA	Continuous	Blomberg's K	110	0.1269	0.058+	
Height	Continuous	Blomberg's K	88	0.36	0.007**	
Leaf toughness	Continuous	Blomberg's K	105	0.0227	0.25	
Wood density	Continuous	Blomberg's K	33	0.6495	0.01*	
Circular traits						
Flower Period	Circular	Mantel Test	116	0.0127	0.293	
Growth forms						
Stemmed Herb	Binary	Phylogenetic D	116	-0.1232	0.686	0***
Tree	Binary	Phylogenetic D	116	-0.2869	0.783	0***
Rosette	Binary	Phylogenetic D	116	-0.3921	0.89	0***
Vine	Binary	Phylogenetic D	116	0.8329	0.03*	0.297
SubShrub	Binary	Phylogenetic D	116	-4.2224	0.663	0.274
Shrub	Binary	Phylogenetic D	116	0.1686	0.411	0.036*
Elongated Leafy Rhizomatous	Binary	Phylogenetic D	116	-0.2569	0.679	0.001***
Nitrogen Fixer	Binary	Phylogenetic D	116	-1.8579	1	0***
Dispersal mechanisms						
Subterranean	Binary	Phylogenetic D	116	-1.5832	0.54	0.347
Unassisted	Binary	Phylogenetic D	116	0.7722	0***	0.075+
Wind	Binary	Phylogenetic D	116	0.468	0.027*	0***
ExoZoochory	Binary	Phylogenetic D	116	0.546	0.045*	0.017*
Ballistic	Binary	Phylogenetic D	116	0.1844	0.36	0.001***
EndoZoochory	Binary	Phylogenetic D	116	0.1859	0.241	0***
Hoarding	Binary	Phylogenetic D	116	-0.8997	0.81	0.007**
Myrmecochory	Binary	Phylogenetic D	116	1.606	0.06+	0.77
Water	Binary	Phylogenetic D	116	0.613	0.107	0.127
All traits combined	Gower Distance Matrix	Mantel Test	116	0.2475	0.001***	

Table S3.3: Results of tests for phylogenetic signal in our functional traits using the Genbank-only tree. Significance levels for test statistics: *** $p \leq 0.001$, ** $p \leq 0.01$, * $p \leq 0.05$, + $p \leq 0.1$

	Data type	*Test*	*N*	*Value*	*p-value Brownian Motion*	*p-value random*
Continuous traits						
SLA	Continuous	Blomberg's K	110	0.1066	0.06+	
Height	Continuous	Blomberg's K	88	0.3567	0.014*	
Leaf toughness	Continuous	Blomberg's K	105	0.0224	0.259	
Wood density	Continuous	Blomberg's K	33	0.6495	0.007**	
Circular traits						
Flower Period	Circular	Mantel Test	112	0.0143	0.259	
Growth forms						
Stemmed Herb	Binary	Phylogenetic D	116	-0.2699	0.862	0***
Tree	Binary	Phylogenetic D	116	-0.2391	0.74	0***
Rosette	Binary	Phylogenetic D	116	-0.5285	0.929	0***
Vine	Binary	Phylogenetic D	116	0.9297	0.007**	0.412
SubShrub	Binary	Phylogenetic D	116	-5.0503	0.649	0.289
Shrub	Binary	Phylogenetic D	116	0.1807	0.406	0.034*
Elongated Leafy Rhizomatous	Binary	Phylogenetic D	116	-0.1693	0.641	0.001***
Nitrogen Fixer	Binary	Phylogenetic D	116	-1.9747	1	0***
Dispersal mechanisms						
Subterranean	Binary	Phylogenetic D	116	-2.8884	0.605	0.306
Unassisted	Binary	Phylogenetic D	116	0.7219	0.002**	0.045*
Wind	Binary	Phylogenetic D	116	0.3379	0.08+	0***
ExoZoochory	Binary	Phylogenetic D	116	0.4296	0.082+	0.01**
Ballistic	Binary	Phylogenetic D	116	0.2836	0.236	0.005**
EndoZoochory	Binary	Phylogenetic D	116	0.1559	0.277	0***
Hoarding	Binary	Phylogenetic D	116	-0.6596	0.745	0.01**
Myrmecochory	Binary	Phylogenetic D	116	0.8581	0.178	0.426
Water	Binary	Phylogenetic D	116	0.8541	0.039*	0.341
All traits combined	Gower Distance Matrix	Mantel Test	112	0.2578	0.001***	

Table S3.4: Mean community coverage for each trait across all communities. Dispersal includes all *Dispersal mechanisms* from tables S2.1-3, and Growth_Form includes all *Growth forms* from Tables S2.1-3.

Trait	Average Coverage Across All Communities
Clonal	0.85748
Dispersal	0.924648
Flower_Period	0.924648
Growth_Form	0.924648
Height	0.794681
SLA	0.916164
Tough	0.81557
Wood_Density	0.287791
Woody	0.924648

Fig S3.2 – 11: The following figures show results for selected combinations of functional trait distinctiveness used as predictors of the effect size of competition (i.e. when a = 0 in main text Eq. 2). Grey lines showing the predicted effect size of competition are shown only when the overall model is significant. Due to the number of possible combinations, only combinations which yielded at least one significant model are shown.

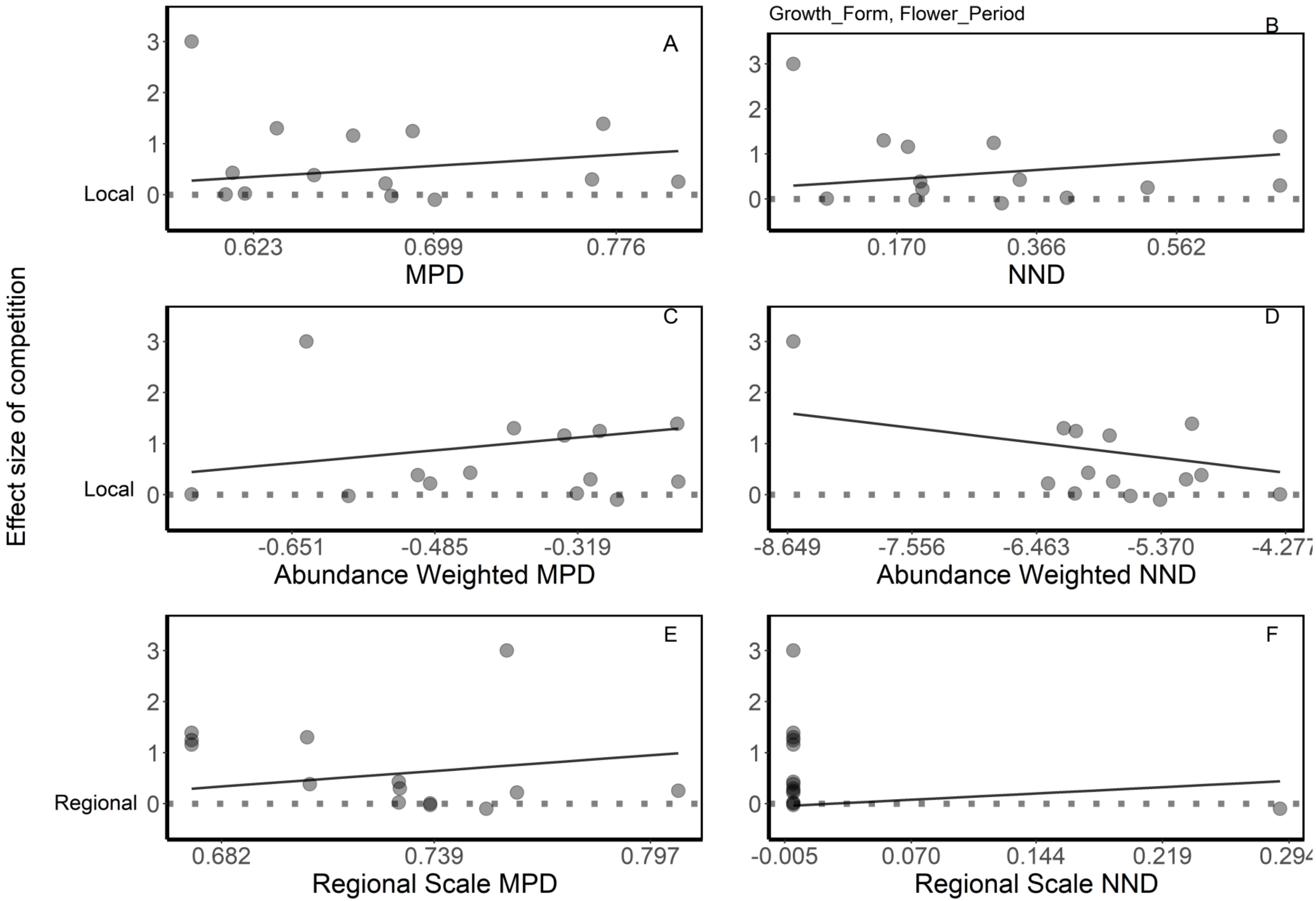
Growth_Form, Flower_Period
Effect size of competition
A
Local
MPD
0.623
0.699
0.776
B
NND
0.170
0.366
0.562
C
Local
Abundance Weighted MPD
-0.651
-0.485
-0.319
D
Abundance Weighted NND
-8.649
-7.556
-6.463
-5.370
-4.277
E
Regional
Regional Scale MPD
0.682
0.739
0.797
F
Regional Scale NND
-0.005
0.070
0.144
0.219
0.294
0
1
2
3

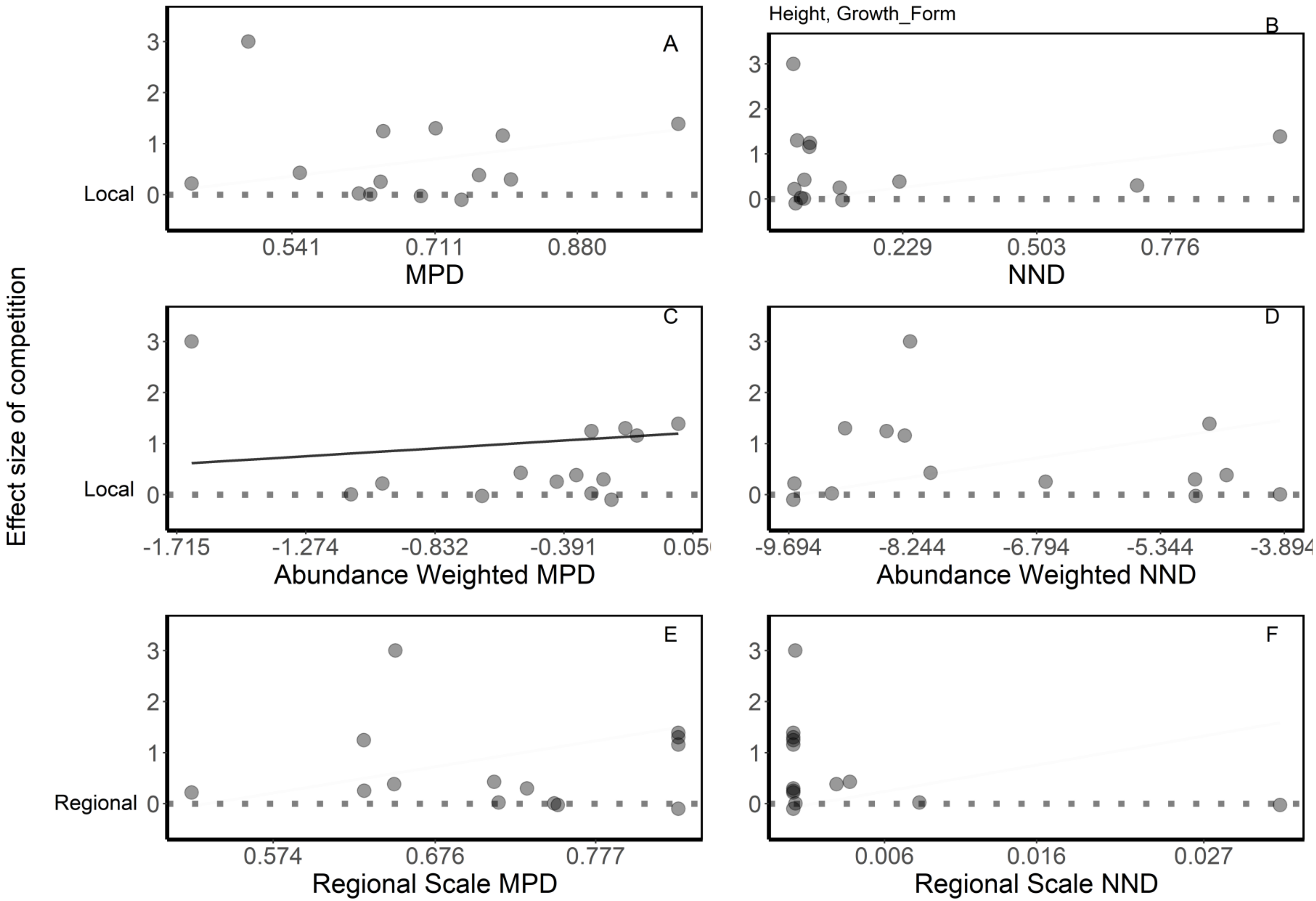

Effect size of competition
Height, Growth_Form
A
B
C
D
E
F
Local
Local
Regional
MPD
NND
Abundance Weighted MPD
Abundance Weighted NND
Regional Scale MPD
Regional Scale NND
0.541
0.711
0.880
0.229
0.503
0.776
-1.715
-1.274
-0.832
-0.391
-9.694
-8.244
-6.794
-5.344
0.574
0.676
0.777
0.006
0.016
0.027

188

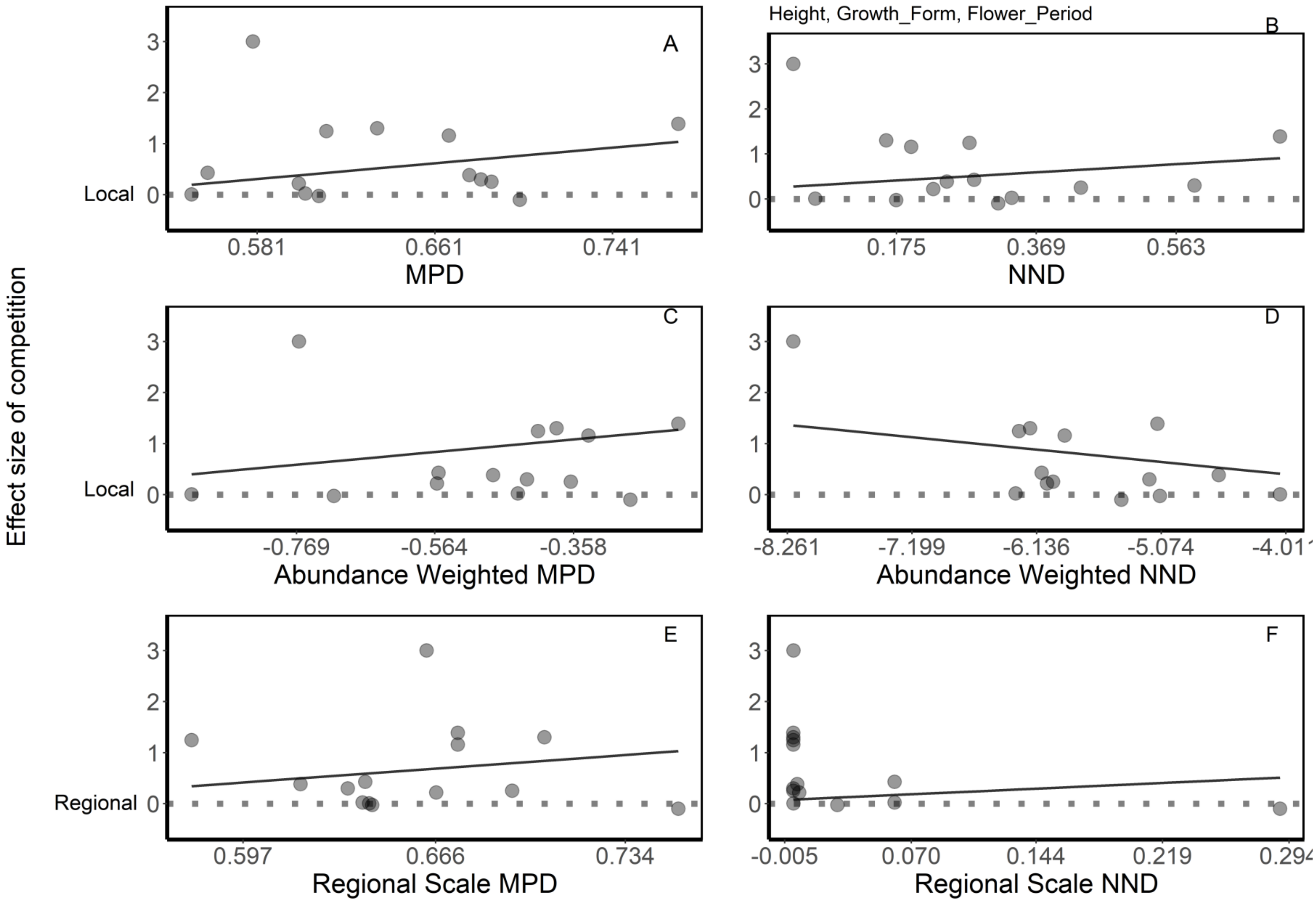

Height, Growth_Form, Flower_Period
Effect size of competition
A
B
C
D
E
F
Local
Local
Regional
MPD
NND
Abundance Weighted MPD
Abundance Weighted NND
Regional Scale MPD
Regional Scale NND
0.581
0.661
0.741
0.175
0.369
0.563
-0.769
-0.564
-0.358
-8.261
-7.199
-6.136
-5.074
0.597
0.666
0.734
-0.005
0.070
0.144
0.219

189

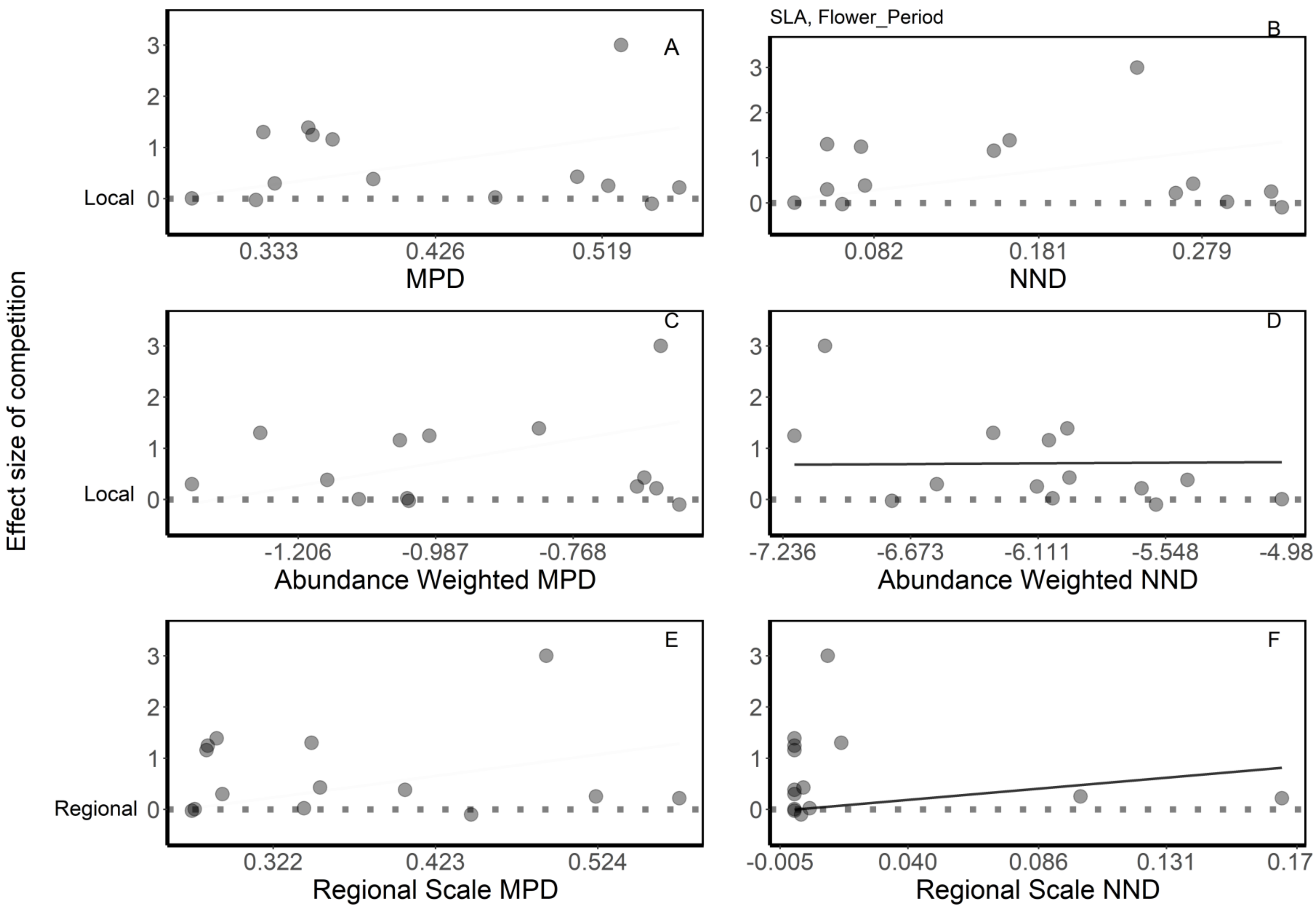
SLA, Flower_Period
Effect size of competition
A
B
C
D
E
F
Local
Local
Regional
0
1
2
3
0.333
0.426
0.519
MPD
0.082
0.181
0.279
NND
-1.206
-0.987
-0.768
Abundance Weighted MPD
-7.236
-6.673
-6.111
-5.548
-4.98
Abundance Weighted NND
0.322
0.423
0.524
Regional Scale MPD
-0.005
0.040
0.086
0.131
0.17
Regional Scale NND

191

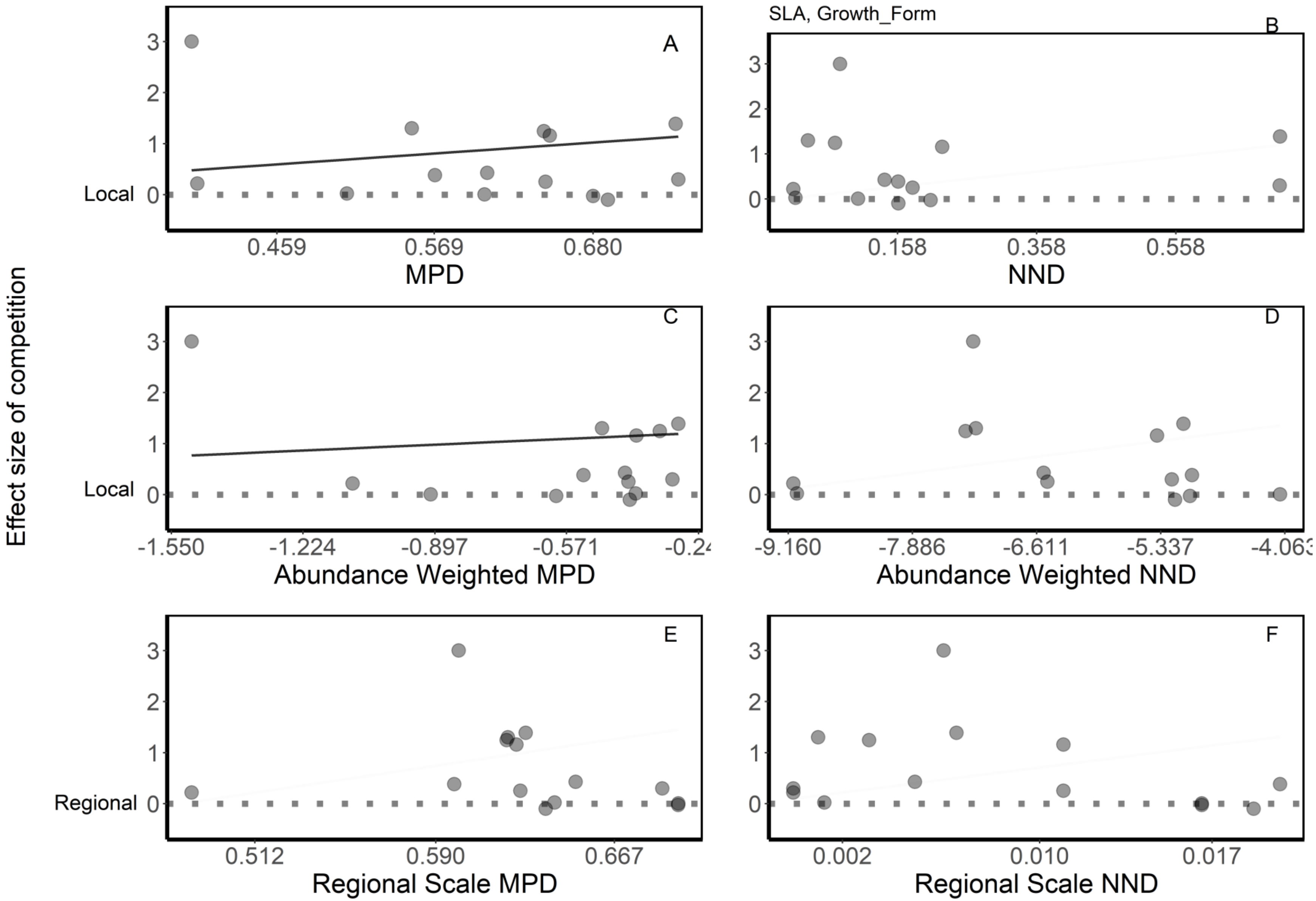
Effect size of competition
SLA, Growth_Form
A
B
C
D
E
F
Local
Local
Regional
MPD
NND
Abundance Weighted MPD
Abundance Weighted NND
Regional Scale MPD
Regional Scale NND
0.459
0.569
0.680
0.158
0.358
0.558
-1.550
-1.224
-0.897
-0.571
-9.160
-7.886
-6.611
-5.337
0.512
0.590
0.667
0.002
0.010
0.017

192

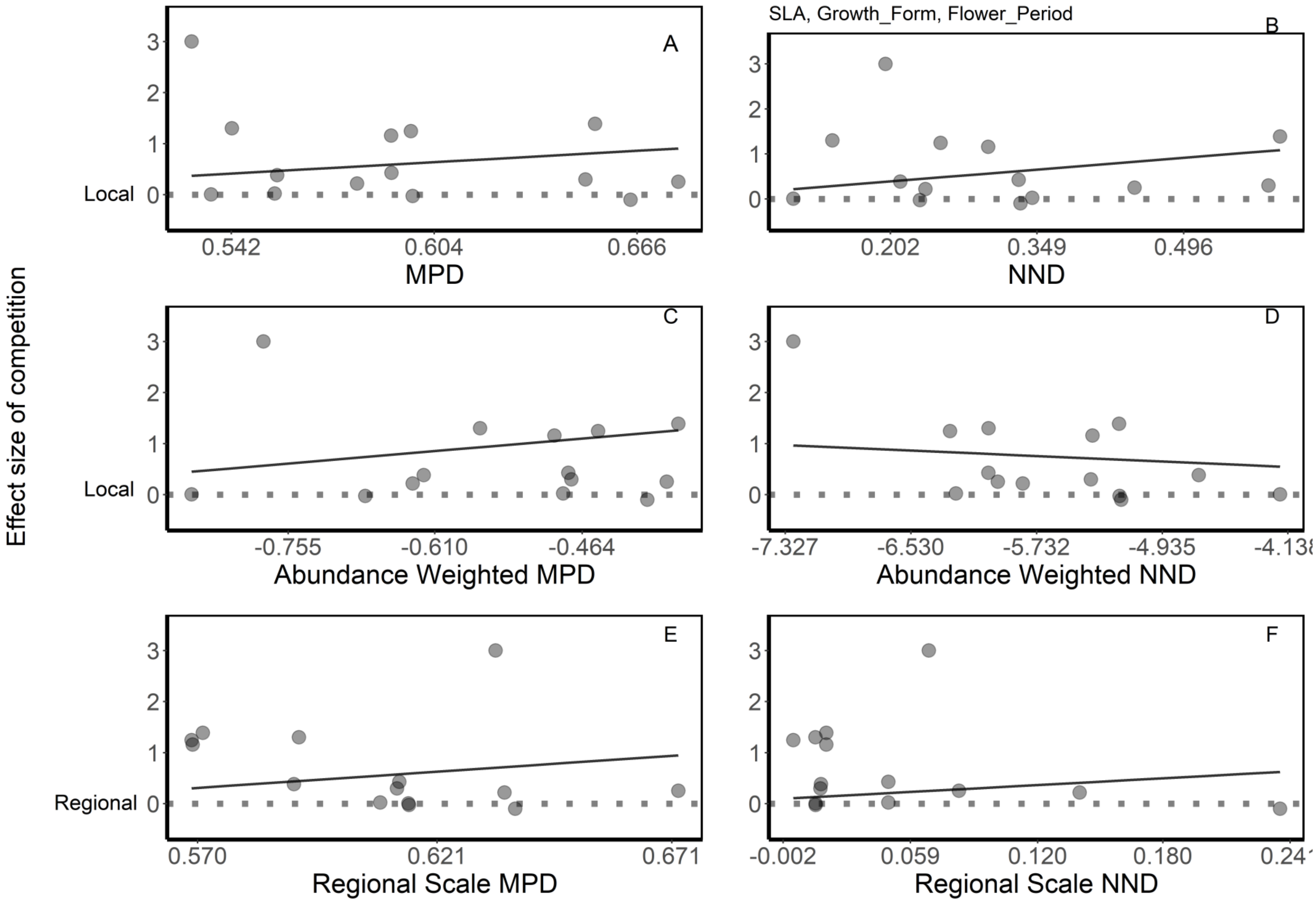
Effect size of competition
Local
Regional
SLA, Growth_Form, Flower_Period
A
B
C
D
E
F
MPD
NND
Abundance Weighted MPD
Abundance Weighted NND
Regional Scale MPD
Regional Scale NND
0.542
0.604
0.666
0.202
0.349
0.496
-0.755
-0.610
-0.464
-7.327
-6.530
-5.732
-4.935
0.570
0.621
0.671
-0.002
0.059
0.120
0.180

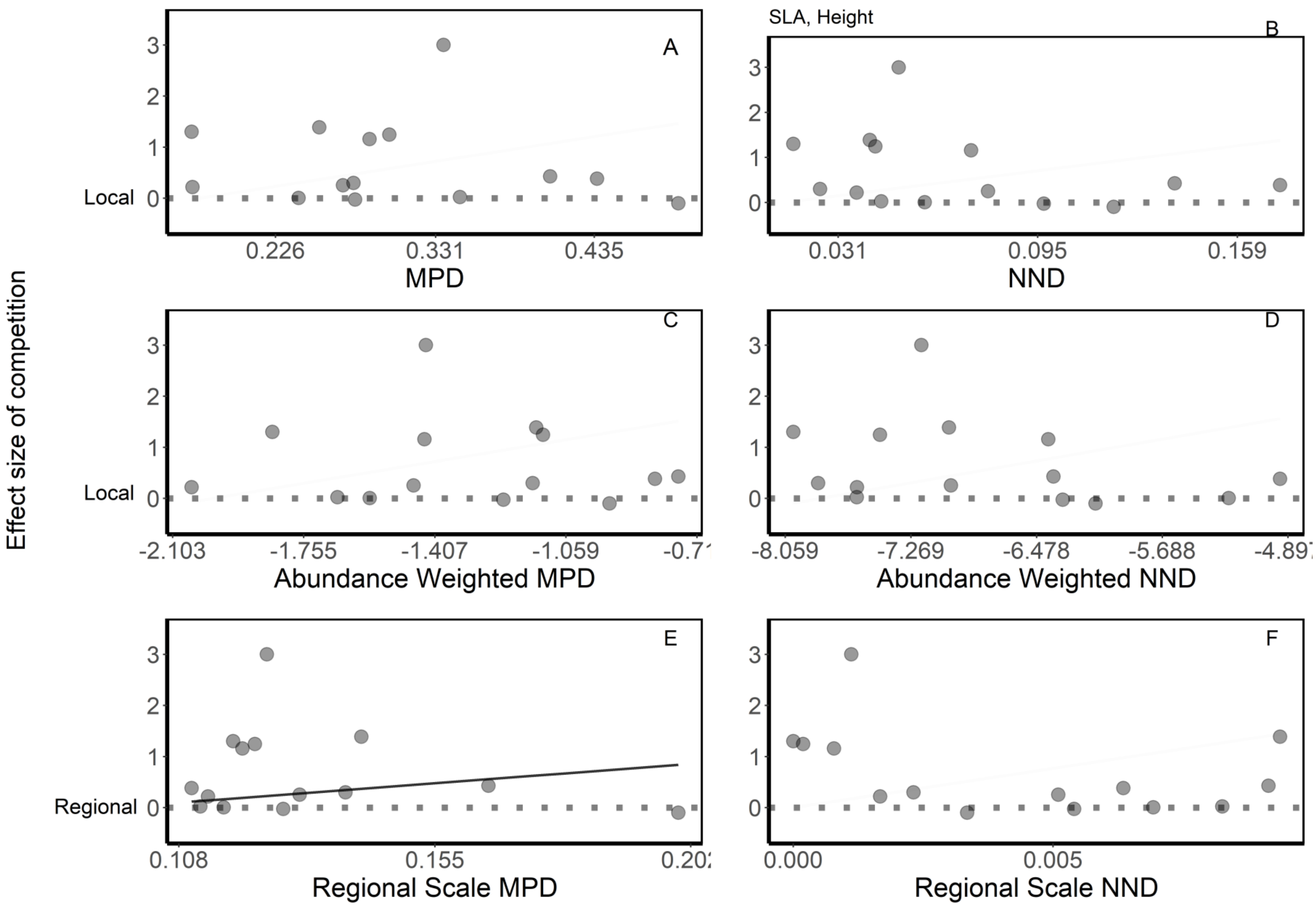

Effect size of competition
SLA, Height
A
B
C
D
E
F
Local
Local
Regional
MPD
NND
Abundance Weighted MPD
Abundance Weighted NND
Regional Scale MPD
Regional Scale NND

193

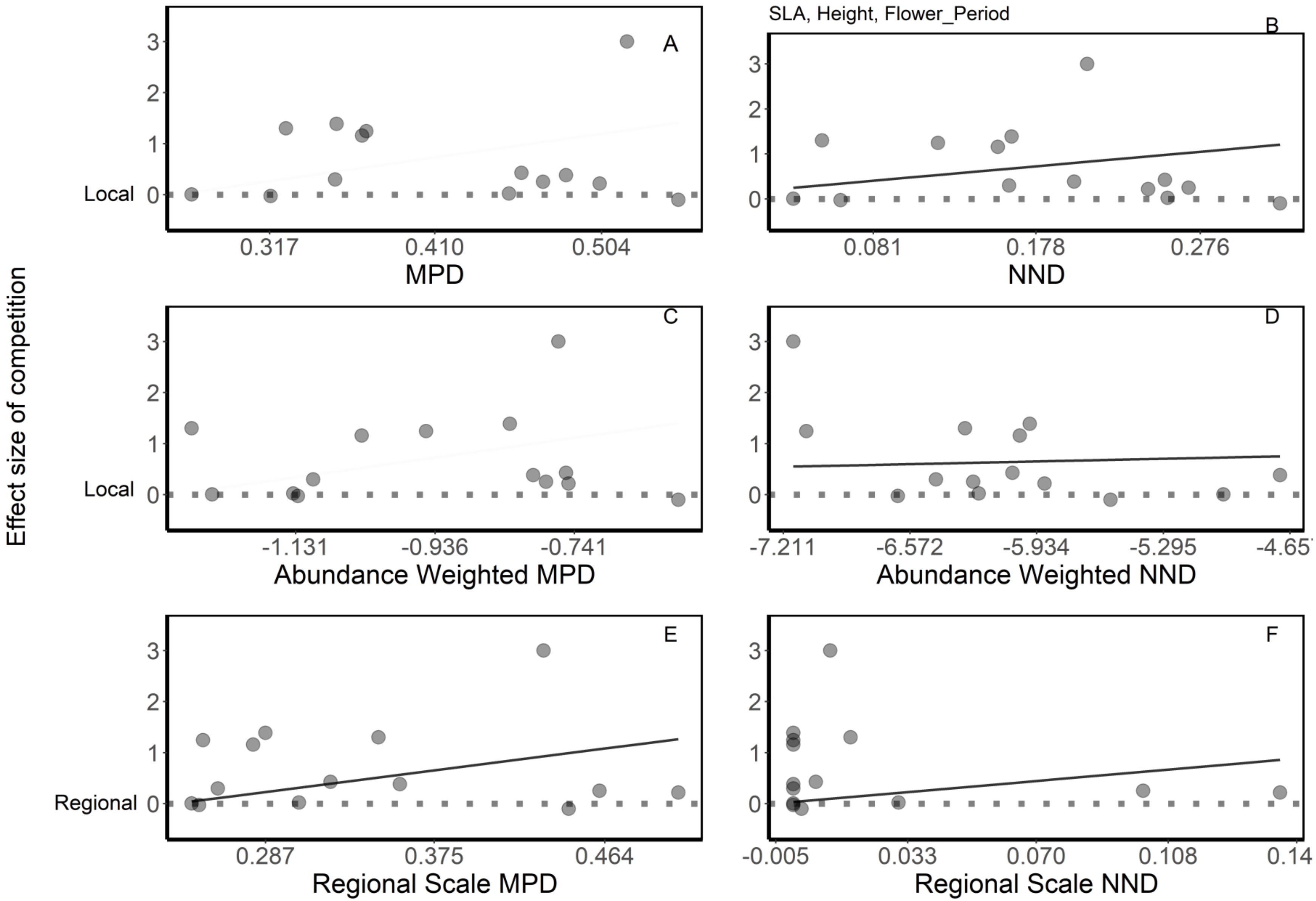
SLA, Height, Flower_Period
Effect size of competition
Local
Regional
A
B
C
D
E
F
MPD
NND
Abundance Weighted MPD
Abundance Weighted NND
Regional Scale MPD
Regional Scale NND

194

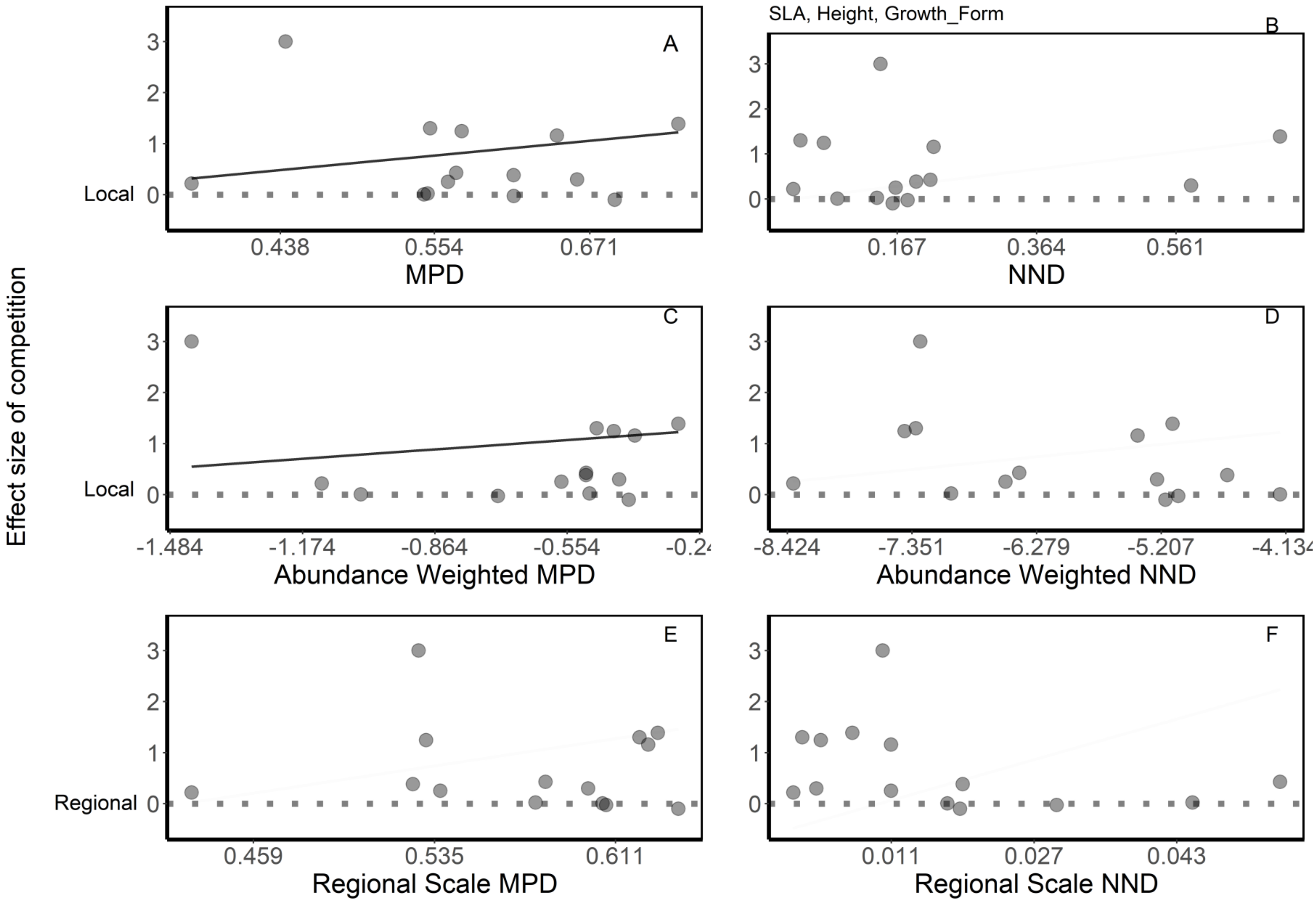

SLA, Height, Growth_Form
A
B
C
D
E
F
Effect size of competition
Local
Local
Regional
0.438
0.554
0.671
MPD
0.167
0.364
0.561
NND
-1.484
-1.174
-0.864
-0.554
Abundance Weighted MPD
-8.424
-7.351
-6.279
-5.207
Abundance Weighted NND
0.459
0.535
0.611
Regional Scale MPD
0.011
0.027
0.043
Regional Scale NND

195

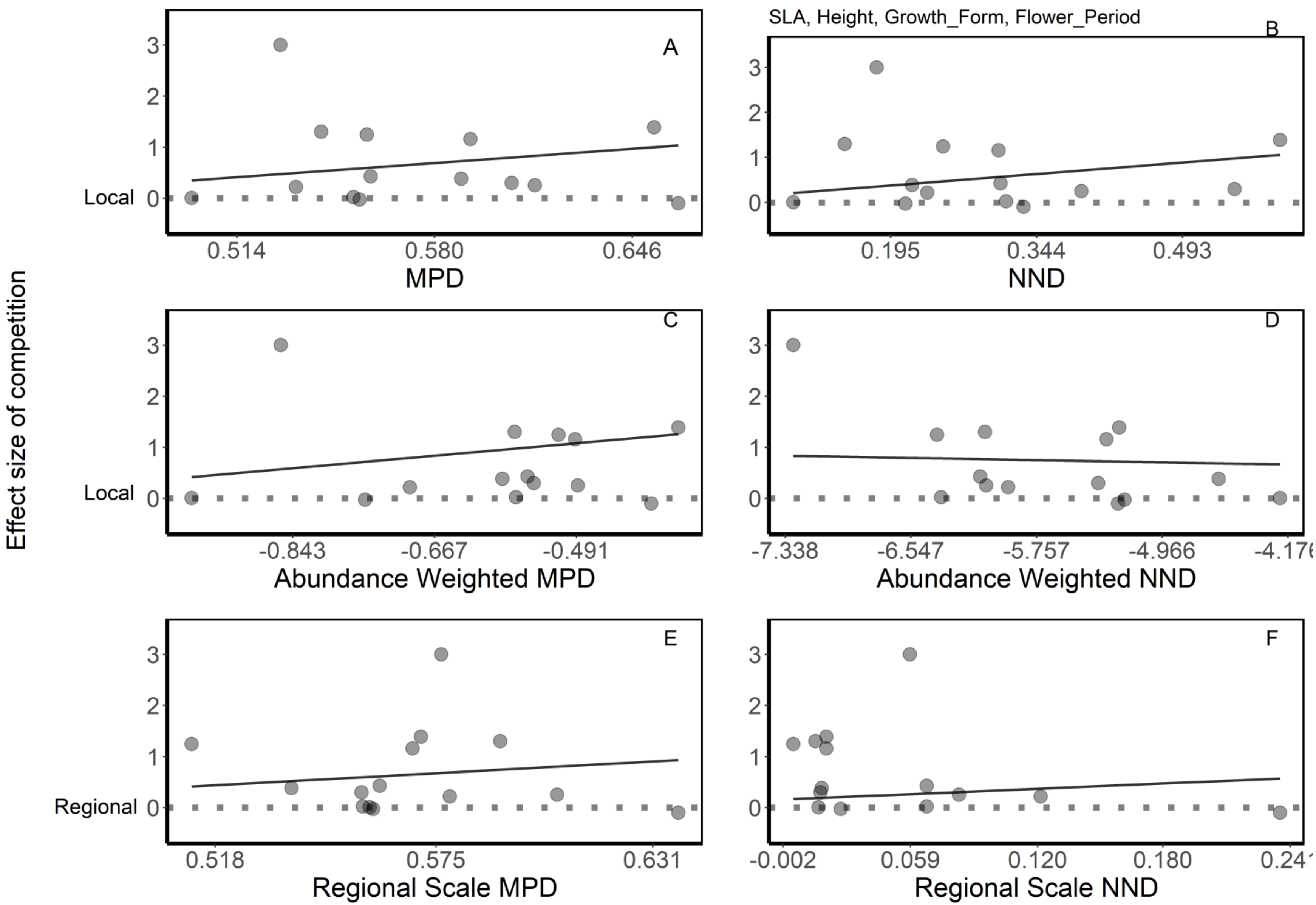

SLA, Height, Growth_Form, Flower_Period
Effect size of competition
A
Local
MPD
0.514
0.580
0.646
B
NND
0.195
0.344
0.493
C
Local
Abundance Weighted MPD
-0.843
-0.667
-0.491
D
Abundance Weighted NND
-7.338
-6.547
-5.757
-4.966
E
Regional
Regional Scale MPD
0.518
0.575
0.631
F
Regional Scale NND
-0.002
0.059
0.120
0.180

196

197

Literature

Perez-Harguindeguy N., Diaz S., Garnier E., Lavorel S., Poorter H., Jaueguiberry P., Bret-Harte M.S. *et al*. (2013). New handbook for standardized measurement of plant functional traits worldwide. *Aus J. Bot.* 61, 167-234.

Pretzsch H. & Biber P. (2010) Size-symmetric versus size-asymmetric competition and growth partitioning among trees in forest stands along an ecological gradient in central Europe. *Can. J. For. Res.* 40, 370-384. DOI: 10.1139/X09-195

Spasojevic, M.J., Turner, B.L. & Myers, J.A. (2016). When does intraspecific trait variation contribute to functional beta-diversity? *J. Ecol.,* 104, 487-496.

Weiner J., Stoll P., Muller-Landau H. & Jasentuliyana A. (2001). The effects of density, spatial pattern, and competitive symmetry on size variation in simulated plant populations. *Am. Nat.*158(4), 438-459.
